# Frankincense essential oil prepared from hydrodistillation of *Boswellia sacra* gum resins induces human pancreatic cancer cell death in cultures and in a xenograft murine model

**DOI:** 10.1186/1472-6882-12-253

**Published:** 2012-12-13

**Authors:** Xiao Ni, Mahmoud M Suhail, Qing Yang, Amy Cao, Kar-Ming Fung, Russell G Postier, Cole Woolley, Gary Young, Jingzhe Zhang, Hsueh-Kung Lin

**Affiliations:** 1 Department of General Surgery, Long Hua Hospital Shanghai University of Traditional Chinese Medicine, Shanghai, China; 2 Al Afia Medical Complex, Salalah, Sultanate of Oman; 3 Department of Urology, University of Oklahoma Health Sciences Center, Oklahoma City, OK, 73104, USA; 4 Department of Biological Sciences, University of Southern California, Los Angeles, CA, 90089, USA; 5 Department of Pathology, University of Oklahoma Health Sciences Center, Oklahoma City, OK, 73104, USA; 6 Oklahoma City Veterans Medical Center, Oklahoma City, OK, 73104, USA; 7 Department of Surgery, University of Oklahoma Health Sciences Center, Oklahoma City, OK, 73104, USA; 8 Young Living Essential Oils, Lehi, UT, 84043, USA

**Keywords:** Apoptosis, *Boswellia sacra*, Boswellic acid, Essential oil, Frankincense, Hydrodistillation, Pancreatic cancer

## Abstract

**Background:**

Regardless of the availability of therapeutic options, the overall 5-year survival for patients diagnosed with pancreatic cancer remains less than 5%. Gum resins from *Boswellia* species, also known as frankincense, have been used as a major ingredient in Ayurvedic and Chinese medicine to treat a variety of health-related conditions. Both frankincense chemical extracts and essential oil prepared from *Boswellia* species gum resins exhibit anti-neoplastic activity, and have been investigated as potential anti-cancer agents. The goals of this study are to identify optimal condition for preparing frankincense essential oil that possesses potent anti-tumor activity, and to evaluate the activity in both cultured human pancreatic cancer cells and a xenograft mouse cancer model.

**Methods:**

*Boswellia sacra* gum resins were hydrodistilled at 78°C; and essential oil distillate fractions were collected at different durations (Fraction I at 0–2 h, Fraction II at 8–10 h, and Fraction III at 11–12 h). Hydrodistillation of the second half of gum resins was performed at 100°C; and distillate was collected at 11–12 h (Fraction IV). Chemical compositions were identified by gas chromatography–mass spectrometry (GC-MS); and total boswellic acids contents were quantified by high-performance liquid chromatography (HPLC). Frankincense essential oil-modulated pancreatic tumor cell viability and cytotoxicity were determined by colorimetric assays. Levels of apoptotic markers, signaling molecules, and cell cycle regulators expression were characterized by Western blot analysis. A heterotopic (subcutaneous) human pancreatic cancer xenograft nude mouse model was used to evaluate anti-tumor capability of Fraction IV frankincense essential oil *in vivo*. Frankincense essential oil-induced tumor cytostatic and cytotoxic activities in animals were assessed by immunohistochemistry.

**Results:**

Longer duration and higher temperature hydrodistillation produced more abundant high molecular weight compounds, including boswellic acids, in frankincense essential oil fraactions. Human pancreatic cancer cells were sensitive to Fractions III and IV (containing higher molecular weight compounds) treatment with suppressed cell viability and increased cell death. Essential oil activated the caspase-dependent apoptotic pathway, induced a rapid and transient activation of Akt and Erk1/2, and suppressed levels of cyclin D1 cdk4 expression in cultured pancreatic cancer cells. In addition, *Boswellia sacra* essential oil Fraction IV exhibited anti-proliferative and pro-apoptotic activities against pancreatic tumors in the heterotopic xenograft mouse model.

**Conclusion:**

All fractions of frankincense essential oil from *Boswellia sacra* are capable of suppressing viability and inducing apoptosis of a panel of human pancreatic cancer cell lines. Potency of essential oil-suppressed tumor cell viability may be associated with the greater abundance of high molecular weight compounds in Fractions III and IV. Although chemical component(s) responsible for tumor cell cytotoxicity remains undefined, crude essential oil prepared from hydrodistillation of *Boswellia sacra* gum resins might be a useful alternative therapeutic agent for treating patients with pancreatic adenocarcinoma, an aggressive cancer with poor prognosis.

## Background

Pancreatic cancer is one of the most common cancers, ranking the fourth most common cause of cancer*-*related mortality in the United States [1] and across the world [2]. Due to the lack of specific and effective biomarkers for early detection and diagnosis, the majority of cases (53%) are diagnosed when cancer has already metastasized. Based on Surveillance, Epidemiology, and End Results (SEER) data for 2002–2008, the overall 5-year relative survival was 5.8%, with a survival rate of 23.3% for patients with localized disease and 1.8% for those with distant metastases ( http://seer.cancer.gov/statfacts/html/pancreas.html). Gemcitibine (Gemzar®), 5-flourouracil, and erlotinib have been used as therapeutic agents for treating advanced pancreatic cancer; however, these agents are associated with poor responses, multiple adverse events, and drug resistance [3-6]. There is a vital need to identify and develop novel therapeutic agents that will specifically and effectively target pancreatic cancer cells.

Aromatic gum resins obtained from trees of the genus *Boswellia* (family Burseraceae), also known as frankincense, have been shown to possess anti-tumor activity. Winking *et al*. reported that a frankincense extract induces apoptosis and prolong survival in a rat glioma model [7]. A methanol extract of *Boswellia serrata* inhibits abnormal skin cell proliferation induced by 12-O-tetradecanoylphorbol-13-acetate (TPA) and tumor promotion initiated by 7,12-dimethylbenz[a]anthracene (DMBA) in a mouse model [8]. In a human clinical study, a *Boswellia serrata* resin extract has been shown to reduce cerebral edema and potential anti-cancer activity in patients irradiated for brain tumors [9]. These studies suggest that gum resins of *Boswellia* species contain active ingredients that have anti-cancer activity.

We previously reported that cultured human bladder and breast cancer cells are more sensitive to frankincense essential oils prepared from both *Boswellia carteri* and *Boswellia sacra* than their normal counterparts with suppressed proliferation and increased apoptosis [10,11]. The anti-cancer activity is mediated through multiple signaling pathways. In addition, frankincense essential oil overcomes multicellular resistant and invasive phenotypes of human breast cancer cells. Using frankincense essential oil obtained from hydrodistillation of *Boswellia sacra* gum resins, our goals are to determine optimal preparation conditions that induce potent cytotoxic effects in cultured human pancreatic cancer cells, to establish a relationship between *Boswellia sacra* essential oil chemical composition and anti-cancer activity, and to evaluate *Boswellia sacra* essential oil anti-tumor activity *in vivo*. Our results demonstrated that anti-tumor activity of *Boswellia sacra* essential oil depends upon hydrodistillation duration and hydrodistillation temperature; and high molecular weight compounds in the essential oil may be responsible for its anti-tumor properties. More importantly, frankincense essential oil-activated anti-tumor activity was observed in both *in vitro* and *in vivo* conditions.

## Methods

### Reagents and chemicals

Cell culture media (DMEM and RPMI 1640), fetal bovine serum (FBS), sodium pyruvate, and penicillin-streptomycin were purchased from Invitrogen (Grand Island, NY). XTT cell proliferation assay, lactate dehydrogenase (LDH) cytotoxicity detection, and *in situ* cell death detection kits were obtained from Roche Applied Science (Indianapolis, IN). Bicinchoninic acid (BCA) protein assay kit was purchased from Thermo Scientific Pierce (Rockford, IL). Rabbit anti-phospho-Akt (protein kinase B; PKB) (Ser473) antibody, rabbit anti-phospho-p44/42 MAP kinase (ERK1/2) (Thr202/Tyr204) antibody, mouse anti-cyclin D1 monoclonal antibody, mouse anti-cdk4 monoclonal antibody, mouse anti-human caspase-8 monoclonal antibody, rabbit anti-human caspase-9 polyclonal antibody, rabbit anti-cleaved caspase-3 (Asp175) monoclonal antibody, rabbit anti-poly (ADT-ribose) polymerase (PARP) polyclonal antibody, and rabbit anti-human phospho-histone H3 (PHH3) (Ser10) polyclonal antibody were purchased from Cell Signaling Technology (Danvers, MA). Mouse anti-human pro-caspase-3 monoclonal antibody was obtained from abcam (Cambridge, MA). Mouse anti-β-actin antibody was obtained from Sigma (St. Louis, MO). Matrigel™ basement membrane matrix was purchased from BD Biosciences (Bedford, MA).

### Frankincense essential oil preparation

Hougari grade *Boswellia sacra* resins were harvested in the Hasik area east of Salalah, Oman. Distillation was performed in a custom made, 250 L-capacity hydrodistiller following previously reported procedures [11]. Four fractions of frankincense essential oils were obtained: 78°C for 0–2 h (Fraction I), 78°C for 8–10 h (Fraction II), 78°C for 11–12 h (Fraction III), and 100°C for 11–12 h (Fraction IV).

### Analysis of chemical components

Preparations and conditions for chemical analysis of *Boswellia sacra* essential oil Fractions I-IV using gas chromatography–mass spectrometry (GC-MS) were the same as reported previously [11]. In addition, the use of high-performance liquid chromatography (HPLC) analysis for boswellic acids quantification in Fractions I-IV essential oils were the same as reported previously [11].

### Human pancreatic cancer cell lines

Four human pancreatic cancer cell lines, MIA PaCa-2, Panc-28, BxPC-3, and DANG, were provided by Dr. Danny Dhanasekaran at the University of Oklahoma Health Sciences Center (Oklahoma City, OK). The MIA PaCa-2 cell line was established from carcinoma of the pancreas [12]. Panc-28 cells were derived from ductal adenocarcinomas of the pancreas [13]. BxPC-3 cells were established from a histologically confirmed adenocarcinoma of the body of the pancreas [14]. DANG is a malignant human pancreatic ductal carcinoma cell line [15]. MIA PaCa-2, Panc-28, and BxPC-3 cells were cultured in DMEM supplemented with 10% FBS, 1 mM sodium pyruvate, and 100 units/ml penicillin-100 μg/ml streptomycin. DANG cells were cultured in RPMI 1640 supplemented with 10% FBS, 1% sodium pyruvate, and penicillin-streptomycin. Cells were maintained in a humidified cell incubator at 37°C and 5% CO_2_ and passaged every 3–4 days or when cells reached 70-80% confluence.

### Cell viability assay

Frankincense essential oil-suppressed human pancreatic tumor cell viability was performed using a colorimetric XTT cell proliferation assay kit. Human pancreatic cancer cell seeding, frankincense essential oils treatment (1:200 to 1:2,700 dilutions), and XTT assay were performed as reported previously [10,11]. Numbers of viable cells were calculated from standard curves with known numbers of cells run in parallel. Cell viability was calculated by dividing the number of viable cells at 24 h after essential oil treatment by the number of cells at the time of treatment. Results were presented as percentages of cell viability.

### Cell cytotoxicity assay

Frankincense essential oil-induced damage in pancreatic cancer cells was quantified using the LDH cytotoxicity detection reagent. Conditions for cell seeding and essential oil treatment were identical those reported previously [11]. Numbers of damaged cells were calculated from standard curves with cell lysis buffer-induced cell death. Cell cytotoxicity was calculated by dividing the number of damaged cells in essential oil-treated wells by total numbers of cells in untreated wells determined by the XTT assay. Results were presented as percentages of cell death.

### Genomic DNA fragmentation

To determine whether frankincense essential oil induces chromosomal DNA fragmentation (a biochemical hallmark of apoptosis) in tumor cells, 3 × 10^5^ pancreatic cancer cells were seeded in 60 mm tissue culture plates in their growth media, incubated overnight for adherence, and treated with Fraction III (1:600 dilution) or Fraction IV (1:1,200 dilution) essential oil in growth media based on IC50 and LC50 values from all cell lines. Cells were harvested at 0 (untreated control), 1, 2, 4, and 8 h following treatment; and genomic DNA was prepared based on reported procedures [16]. Agarose gel separation of the isolated genomic DNA, gel staining, and gel image capture were performed as described previously [11].

### Western blot analysis

To determine frankincense essential oil-regulated expression of caspases, signaling molecules, and cell cycle regulators, pancreatic cancer cells (5 × 10^5^) were seeded in 60 mm tissue culture plates for adherence and treated with Fraction III (1:600 dilution) or IV (1:1,200 dilution) essential oil. Conditions for total cellular protein isolation and quantification, protein separation using Tris–HCl gels and transfer to PVDF membranes, primary and secondary antibodies incubation, as well as detection of immunoreactive proteins and image capture of the immunoreactive bands followed procedures reported previously [11].

### Subcutaneous tumor implantation and frankincense essential oil administration

The animal protocol was approved by Institutional Animal Care and Use Committees (IACUA) at the University of Oklahoma Health Sciences Center and Shanghai University of Traditional Chinese Medicine. Male athymic (nu/nu) mice between 4–5 weeks old (20–25 g) were purchased and kept in individually ventilated cages with 3–5 mice/cage on sawdust. To establish xenograft tumors, 1 × 10^7^ MIA PaCa-2 cells suspended in 250 μl Hank’s balanced salt solution (HBSS) supplemented with 20% Matrigel™ were subcutaneously implanted into the flank of each mouse.

At day 7 after tumor cells implantation, mice were randomly assigned to receive either 100 μl PBS (control) or a mixture of 30 μl Fraction IV frankincense essential oil plus 70 μl PBS through subcutaneous injections. Animals were treated every 4 days; and a total of 3 injections were administered. Tumor diameters were measured *in situ* in two dimensions at 4 days after each treatment, or immediately before the next treatment, using calipers. Tumor volume was calculated as length (the longest dimension) × width^2^ × 0.52 (mm^3^) [17]. Animals were euthanized at day 4 after the third injection; and the tumors were surgically removed. The isolated tumors were weighted and measured in three dimensions with calipers; and tumor volume was calculated as length × width × height × 0.52 (mm^3^) [18]. Tumors were then fixed in 10% formalin and subjected to paraffin embedding for immunohistochemistry.

### Immunohistochemical staining

To access cytostatic activity of frankincense essential oil Fraction IV *in vivo*, tumor sections were stained for PHH3 (Ser10). Since PHH3 (Ser10) can only be detected during mitosis, PHH3 (Ser10)-positive staining provides a quantitative method to measure numbers of tumor cells undergoing cell division. Paraffin blocks were sectioned at 5 μm thick, deparaffinized, and rehydrated. Immunohistochemistry was performed with a citric acid antigen retrieval protocol as previously described [19]. Primary antibody was applied at 1:400 dilution and incubated at 4°C overnight. Diaminobenzidine and hematoxylin were used as chromogen and counter stain, respectively.

Terminal deoxynucleotidyl transferase dUTP nick end labeling (TUNEL) analysis was applied to determine essential oil-induced tumor cell apoptosis. Briefly, paraffin sections were cut at 5 μm thick, deparaffinized, and rehydrated. Apoptotic cells were detected using the *in situ* cell death detection kit. Following the terminal deoxynucleotidyl transferase reaction, labeled nick ends were visualized by alkaline phosphatase-based immunohistochemistry with fast red as substrate as previously described [20]. Stained slides were washed and sealed with an aqueous mounting medium. For each animal, total numbers of PHH3 (Ser10) and TUNEL immunoreactive cells were calculated by counting randomly selected 20 fields at 40x magnification from 2 separate sections. Results were compared between the control (PBS) and frankincense essential oil Fraction IV treatment groups.

### Statistics analysis

The half maximal inhibitory concentration (IC50) and the half lethal concentration (LC50) of frankincense essential oil were calculated from cell viability and death assays, respectively, using the curve fitting function in Sigma Plot (Systac Software, San Jose, CA). Essential oil-mediated cell viability and cytotoxicity were analyzed using the one-way analysis of variance (ANOVA) followed by post hoc Dunnett’s test. For the comparison of tumor volumes as well as tumor cell growth and death between control and frankincense essential oil-treated animals, Student’s *t*-test was performed. Results were considered statistically significant when *P* < 0.05.

## Results

### Chemical profiles of frankincense essential oils

Chemical constituents of *Boswellia sacra* essential oil fractions were dependent on duration and temperature of hydrodistillation. For example, when essential oils collected from 0–2 h (Fraction I), 8–10 h (Fraction II), and 11–12 h (Fraction III) at 78°C were compared, longer distillation produced higher percentages of sesquiterpenes, between *alpha*-copaene and caryophyllene oxide (Table 1). All three fractions were primarily composed of monoterpenes (82.77-90.67%), including *alpha*-thujene, *beta*-pinene, and myrcene. Among the monoterpenes, *alpha*-pinene was the major compound present in all essential oil fractions, ranging from 65.49% to 78.45%. As anticipated, the abundance of *alpha*-pinene decreased with longer and higher temperature distillation due to its highly volatile nature. Compounds such as borneol, dimethyl ether orcinol, *allo*-aromadendrene, *gamma*-cadinene, and caryophyllene oxide were only present in Fraction III essential oil.

**Table 1:** Chemical profiles of frankincense essential oil fractions

	***Boswellia sacra***	**Fractions (%)**			
**Ret. Index**	**Component**	**I**	**II**	**III**	**IV**
755	toluene	0.18	0.16	0.10	0.06
922	unidentified*	1.13	1.05	1.03	0.80
929	*alpha*-thujene	0.87	1.02	0.98	0.93
940	*alpha*-pinene	78.45	76.29	65.49	59.40
953	camphene + verbenene	4.09	4.39	3.42	3.46
973	sabinene	1.41	1.11	2.12	3.63
979	*beta*-pinene	2.16	2.33	2.27	2.38
987	myrcene	2.56	2.82	7.46	5.36
994	*ortho*-methyl anisole	0.16	0.18	0.17	0.35
1004	*alpha*-phellandrene	0.54	0.69	1.20	1.30
1011	*delta*-3-carene	0.03	0.04	0.89	0.09
1018	para-cymene	0.65	0.87	1.24	1.23
1031	limonene	5.55	6.92	8.43	8.99
1039	cis-*beta*-ocimene	0.07	0.10	0.19	0.33
1055	*gamma*-terpinene	0.06	0.10	0.22	0.44
1079	para-cymenene	0.04	0.05	0.05	0.08
1085	*alpha*-terpinolene	0.04	0.06	0.17	0.29
1101	myrcenol	0.05	0.05	0.06	0.11
1112	*alpha*-campholene aldehyde	0.08	0.13	0.30	0.70
1134	trans-pinocarveol	0.07	0.10	0.27	0.77
1138	cis-verbenol	0.07	0.07	0.22	0.46
1146	unidentified**	0.03	0.05	0.12	0.41
1149	pinocamphone	0.02	0.02	0.04	0.11
1153	*alpha*-phellandren-8-ol	0.03	0.04	0.22	0.80
1160	borneol			0.01	0.06
1166	para-cymene-8-ol	0.03	0.04	0.07	0.13
1171	terpinene-4-ol	0.03	0.04	0.14	0.50
1180	*alpha*-terpineol + myrtenal	0.05	0.06	0.12	0.40
1187	myrtenol	0.02	0.02	0.05	0.13
1191	verbenone		0.07	0.15	0.42
1203	cis-carveol	0.01	0.01	0.04	0.10
1243	dimethyl ether orcinol			0.06	0.10
1278	bornyl acetate	0.04	0.05	0.19	0.47
1339	*alpha*-terpenyl acetate	0.01	0.01	0.05	0.15
1391	*alpha*-copaene	0.01	0.01	0.06	0.13
1400	*beta*-elemene	0.03	0.04	0.41	0.94
1437	trans-*beta*-caryophyllene	0.02	0.02	0.28	0.62
1470	*alpha*-humulene	0.03	0.01	0.11	0.17
1477	allo-aromadendrene			0.02	0.06
1485	*gamma*-muurolene		0.01	0.05	0.10
1494	germacrene D			0.02	0.09
1501	*beta*-selinene	0.01	0.02	0.21	0.45
1509	*alpha*-selinene	0.02	0.01	0.12	0.24
1523	*gamma*-cadinene			0.04	0.07
1527	*delta*-cadinene		0.01	0.05	0.14
1593	caryophyllene oxide			0.02	0.05
	**Total**	**99.30**	**99.42**	**99.31**	**98.78**

*unidentified m/z 93(100%), 41(54%), 67(39%), 69(36%), 91(33%), 77(29%), 79(26%), 39(25%), 108(15%), 53(14%), 55(12%), 121(9%).

**unidentified m/z 59(100%), 79(85%), 94(85%), 91(66%), 77(37%), 43(37%), 93(36%), 92(20%), 39(17%), 119(11%), 51(10%), 78(10%).

Frankincense essential oil obtained from 11-12h at 100°C hydrodistillation (Fraction IV) consisted of all compounds detected in Fraction III essential oil. Compounds with higher retention indices, starting with sabinene (RI 973), were present in higher quantities in Fraction IV than in Fraction I, II, or III essential oil with few exceptions (Table 1).

### Boswellic acids contents in frankincense essential oil fractions

Since triterpenes including boswellic acids could not be detected by the described GC-MS protocol used in our laboratory due to their extremely low volatility, an HPLC method was used to determine total boswellic acids contents in the four fractions. We found that boswellic acids contents depended on hydrodistillation duration and temperature (Table 2). Essential oils prepared from longer distillation time and higher distillation temperature contained greater amounts of boswellic acids. For example, boswellic acids contents in Fractions III (19.6%) and IV (30.1%) were higher than those detected in Fraction I (0.9%) or II (0.8%) essential oil.

**Table 2:** Boswellic acids in frankincense essential oil fractions

	**Specific gravity**	**Total boswellic acids (mg/ml)**
Fraction I	0.885	0.91
Fraction II	0.866	0.81
Fraction III	0.852	19.6
Fraction IV	0.847	30.1

### Frankincense essential oil-regulated tumor cell viability

**Figure 1:**
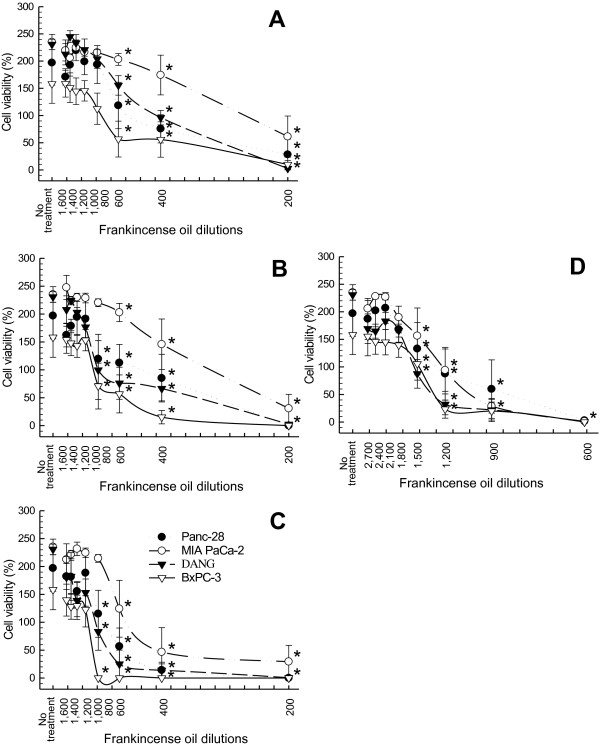
**Suppression of pancreatic cancer cell viability in response to frankincense essential oil treatment.** Human pancreatic cancer cells (5 × 10^3^ in 100 μl) were seeded in each well of 96-well tissue culture plates for adherence. Cells were subjected to serial dilutions of frankincense essential oils treatment, (**A**) Fraction I obtained at 78°C hydrodistillation for 0–2 h, (**B**) Fraction II obtained at 78°C for 8–10 h. (**C**) Fraction III obtained at 78°C for 11–12 h, or (**D**) Fraction IV obtained at 100°C for 11–12 h. All experiments were prepared in triplicate. Cell viability was determined at 24 h following essential oil treatment using the XTT colorimetric assay. Results are presented as mean cell viability (%) ± standard error of mean (SEM) in relation to the number of cells at the time of essential oil treatment from at least 4 independent experiments. * indicates statistical difference in cell viability between essential oil-treated and untreated cells (*P* < 0.05).

All four fractions of essential oils were studied for their potency in suppressing pancreatic cancer cell viability. Based on results from preliminary testing, 1:200 to 1:1,600 dilutions were used for essential oils collected at 78°C (Fractions I-III), and a wider range of dilutions (1:600–1:2,700) was used for Fraction IV essential oil. Although different cancer cell lines had different sensitivities to frankincense essential oils treatment, all fractions, in general, suppressed viability of the human pancreatic cancer cell lines (Figure 1).

Frankincense essential oil-suppressed cell viability was dependent on length and temperature of hydrodistillation. Fractions I and II essential oils were the least potent in suppressing cancer cell viability (Figure 1A and 1B). In contrast, Fraction IV possessed the most potent activity followed by Fraction III essential oil (Figure 1D and 1C). IC50 values supported the observation that longer duration and higher temperature distillation produced more potent frankincense essential oils. For example, IC50 values for MIA PaCa-2 cells were 1:270, 1:330, 1:550 and 1:1,300 dilutions for Fractions I, II, III, and IV essential oils, respectively (Table 3). Since Fractions I and II were not as potent as Fraction III or IV in suppressing tumor cell viability, Fractions III and IV containing higher amounts of sesquiterpenes and boswellic acids were the logical choice for further study and comparison for their anti-tumor properties.

**Table 3:** IC50 and LC50 values of frankincense essential oil fractions on human pancreatic cancer cells

	**Fraction I**	**Fraction II**	**Fraction III**	**Fraction IV**
IC50 values	1:270	1:330	1:600	1:1,200
MIA PaCa-2	1:440	1:590	1:860	1:1,230
Panc-28	1:440	1:700	1:930	1:1,560
DANG	1:570	1:720	1:950	1:1,350
BxPC-3				
LC50 values	ND*	ND	1:240	1:1,310
MIA PaCa-2	ND	ND	1:310	1:1,140
Panc-28	ND	ND	NA**	1:820
DANG	ND	ND	NA	1:700
BxPC-3				

*ND: value was not determined.

**NA: value was not available.

**Figure 2:**
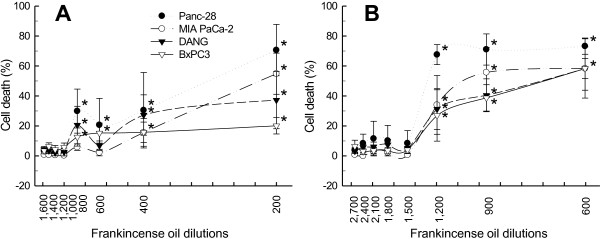
**Quantitative analysis of frankincense essential oil-induced pancreatic cancer cell death.** Human pancreatic cancer cells were seeded into each well of 96-well tissue culture plates at the concentration of 5 × 10^3^/100 μl. After adherence, cells were subjected to serial dilutions of either (**A**) Fraction III or (**B**) Fraction IV essential oil treatment. At 3 h post-treatment, cell viability was determined by the LDH cytotoxicity detection kit. Experiments were prepared in triplicate; and results are presented as mean % of cell death ± SEM in relation to untreated cells from at least 3 independent experiments. * indicates statistical difference of cell death between essential oil-treated and untreated cells (*P* < 0.05).

Frankincense essential oil-suppressed pancreatic cancer cell viability might result from a combination of reduced cell growth and enhanced cell death. LDH release assay was applied to determine frankincense essential oil-induced cell membrane damages and cytotoxicity in tumor cells. Fractions III and IV frankincense essential oils significantly induced cytotoxicity of the human pancreatic cancer cell lines shortly (3 h) after treatment in a concentration dependent manner (Figure 2). Fraction IV was more potent than Fraction III essential oil at inducing cell death. LC50 values were calculated for both Fractions III and IV essential oils, and provided in Table 3. Results also showed that at 3 h following treatment DANG and BxPC-3 cells were more resistant to essential oil-induced death.

### Frankincense essential oil-regulated apoptosis

Genomic DNA fragmentation analysis was performed to demonstrate that essential oil induces apoptosis in human pancreatic cancer cells. Fractions III and IV essential oils at 1:600 and 1:1,200 dilutions, respectively, induced DNA fragmentation in a time-dependent manner in MIA PaCa-2, Panc-28, BxPC-3, and DANG cells within 8 h post-treatment as we observed in human breast cancer cell lines [11].

**Figure 3:**
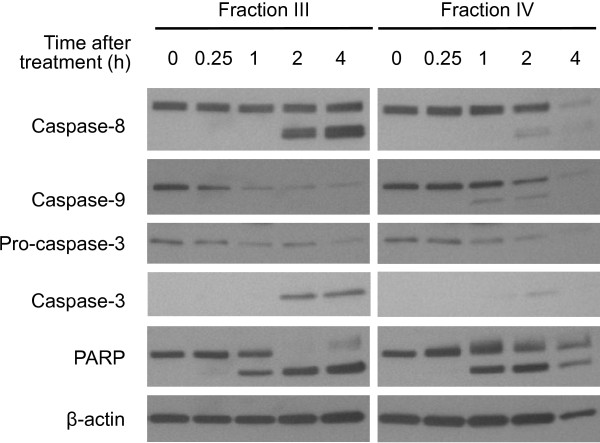
**Frankincense essential oil-induced caspase apoptotic pathway in pancreatic cancer cells.** An aliquot of 5 × 10^5^ pancreatic cancer MIA PaCa-2 cells were seeded in each of 60 mm tissue culture plates. Adherent cells were then subjected to Fractions III (1:600 dilution) or IV (1:1,200 dilution) essential oil stimulation. Total cellular proteins were isolated between 0 (untreated control) and 4 h after treatment. Levels of pro- and activated-caspase-8, 9, 3 and PARP in MIA PaCa-2 cells were detected by Western blot analysis before and after frankincense essential oil treatment. Levels of β-actin was determined in the same samples and used as a protein loading control.

Caspases, a family of aspartate-specific, cysteine proteases, are central components of the machinery responsible for apoptosis. Caspase-2, -8, -9, and −10 have been classified as apoptotic initiator caspases, whereas caspase-3, -6, and −7 are recognized as apoptotic effector caspases. Upon the receipt of death signal, apoptotic initiator caspases are activated (or cleaved) and result in the cleavage of downstream effector caspases. Cleaved caspase-8 p43/p41 and caspase-9 p37/p35 were detected within 2 h in MIA PaCa-2 cells treated with essential oils (Figure 3). Essential oil up-regulated levels of caspase-3 activation corresponding to decreased levels of pro-caspase-3 within 2 h post-stimulation in MIA PaCa-2 cells treated with Fractions III and IV essential oils. Cleavage of PARP, being involved in DNA repair following environmental stress [21] and a main target of activated caspase-3 [22], was also detected in MIA PaCa-2 cells within 1 h following treatment. The other three pancreatic cancer cell lines also exhibited similar patterns of the time-dependent caspases activation. Fraction IV essential oil might induce faster pancreatic cancer cell death as compared to Fraction III, since lower retrievable genomic DNA and total cellular proteins were observed in Fraction IV essential oil (1:1,200 dilution)-treated MIA PaCa-2 cells at 4 h (Figure 3).

### Regulation of signaling and cell cycle molecules by frankincense essential oil

**Figure 4:**
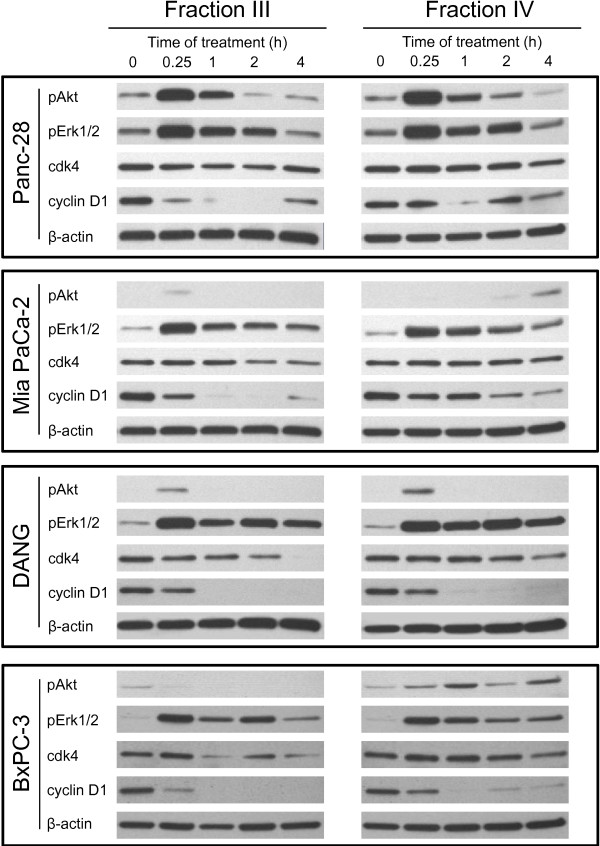
**Frankincense essential oil-regulated signaling and cell cycle-related proteins expression in pancreatic cancer cells.** Human pancreatic cancer cells (5 × 10^5^) were plated into each of 60 mm tissue culture plates for adherence. Cells were treated with either Fractions III (1:600 dilution) or IV (1:1,200 dilution) essential oils. Total cellular proteins were isolated from these cells between 0 (untreated control) and 4 h following essential oil treatment. Levels of Akt and Erk1/2 proteins phosphorylation as well as total cyclin D1 and cdk4 proteins expression were determined using Western blot analysis, and compared between cells with and without frankincense essential oils treatment. Expression of β-actin was also assessed in parallel and used as a protein loading control.

Frankincense essential oil transiently activated both Akt and Erk1/2 signaling molecules in human pancreatic cancer cells within 4 h following treatment. Levels of phospho-Akt Ser(473) expression peaked within 15 min, gradually decreased at 1 h, and returned to untreated levels at 2 h after essential oil exposure in Panc-28 cells (Figure 4). MIA PaCa-2 and DANG cells exhibited transient up-regulation of phospho-Akt Ser(473) within 15 min after treatment, whereas there was no detectible phospho-Akt until 2 h post-stimulation in MIA PaCa-2 cells treated with Fraction IV essential oil. BxPC-3 cells responded differently between Fractions III and IV essential oils; Fraction III essential oil suppressed levels of phospho-Akt Ser(473), whereas Fraction IV up-regulated phosphorylated levels of Akt. In contrast, patterns of frankincense essential oil-stimulated Erk1/2 phosphorylation were similar in these four tumor cell lines. Pancreatic cancer cells responded to essential oils exposures with an immediate increase (within 15 min) followed by gradual decreases (between 1–4 h) in levels of phospho-Erk1/2 (Thr202/Tyr204) expression (Figure 4).

In addition, frankincense essential oil modulated expression of cell cycle regulator proteins, cdk4 and cyclin D1 (crucial cell cycle regulators), in pancreatic cancer cells. Although levels of cdk4 maintained relatively constant in Fractions III and IV essential oils-treated Panc-28 cells within 4 h treatment, cdk4 expression was suppressed following essential oils treatment in MIA PaCa-2, DANG, and BxPC-3 cells (Figure 4). Furthermore, essential oil suppressed cyclin D1 expression almost immediately, within 15 min, after treatment in all four pancreatic cancer cell lines (Figure 4).

### Frankincense essential oil-regulated tumor growth in a xenograft mouse model

Mice bearing human pancreatic cancer MIA PaCa-2 cells were weighted before and after frankincense essential oil treatment. In control animals receiving PBS, averages of body weights increased from 27.53 ± 3.49 g before the first injection to 28.81 ± 2.90, 29.49 ± 3.27, to 30.70 ± 2.85 g at day 4 after the first, second, and third injections, respectively. In contrast, animals received Fraction IV frankincense essential oil treatment maintained similar body weights before (24.93 ± 2.30 g) and 4 days after (24.78 ± 2.57 g) the first injection, but gradually increased to 25.48 ± 2.40 and 26.73 ± 2.67 g at day 4 after the second and third injections, respectively.

**Figure 5:**
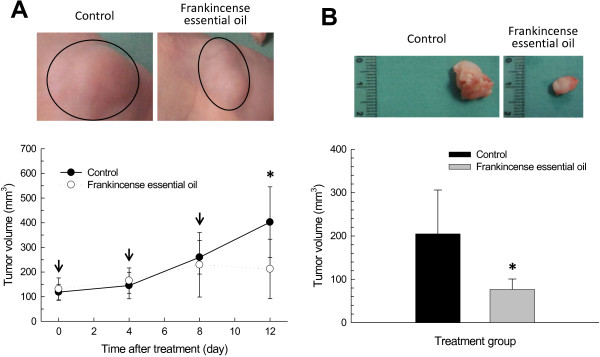
**Frankincense essential oil-suppressed pancreatic tumor growth in a heterotopic xenograft mouse model.** The xenograft mouse tumor model was established by subcutaneous implantation of 1 × 10^7^ MIA PaCa-2 cells in 250 μl HBSS supplemented with 20% Matrigel™ into the flank of each mouse. Tumors were allowed to grow for 7 days. Mice were randomly assigned to receive either 100 μl PBS (control; 5 mice) or Fraction IV frankincense essential oil (30 μl essential oil plus 70 μl PBS; 6 mice). Treatment was administered through subcutaneous injection away from the tumors. (**A**) Tumor volumes measured *in situ*. Arrows indicate the time of tumor size measurement followed by frankincense essential oil treatment. (**B**) Tumor volumes calculated after the tumors have been removed from the experimental subjects at the end of experiments. Images were taken at the time of tumor harvest. * indicates statistical difference between PBS- and essential oil-treated groups (*P* < 0.05).

Tumor volume was measured *in situ* immediately before the beginning of treatment and 4 days after each treatment. Tumors continued to grow in the control group, from 118.6 ± 31.58 mm^3^ before treatment to 402 ± 143.27 mm^3^ at day 12 after the initial treatment (Figure 5A). In contrast, tumors that were treated with Fraction IV essential oil peaked around day 8 (229.50 ± 130.70 mm^3^) and gradually decreased in volumes to 212.50 ± 120.50 mm^3^ at day 12 after the beginning of treatment (Figure 5A). Increases in tumor volume were also calculated in relation to the size at the beginning of treatment; at day 12, tumor volumes increased by 239.32% and 56.78% in the control and frankincense essential oil groups, respectively (Table 4).

**Table 4:** **Changes in tumor volumes (mm**^**3**^**) after frankincense essential oil treatment**

	First injection*	**Second injection**	**Third injection**
Control	20.54±17.31%	124.10±61.67%	239.32±102.25%
Frankincense essential oil Fraction IV	32.73±50.48%	66.54±54.06%	56.78±50.19%

*Tumor volumes were measured on the 4^th^ day after each injection. Changes in tumor volumes after each treatment were calculated based on volumes obtained at the beginning of treatments.

Tumor volume was measured again in three dimensions after they were removed from experimental subjects. Similar to the tumor volume determined *in situ*, significantly smaller tumors were observed in frankincense essential oil-treated animals (76.67 ± 24.11 mm^3^) as compared to the control group (204.80 ± 101.19 mm^3^) (Figure 5B). Although tumor weight in frankincense essential oil-treated group (98.33 ± 41.19 mg) was less than a half of that was observed in the control group (250.00 ± 186.41 mg), the difference was not statistically significant (*P* = 0.082).

**Figure 6:**
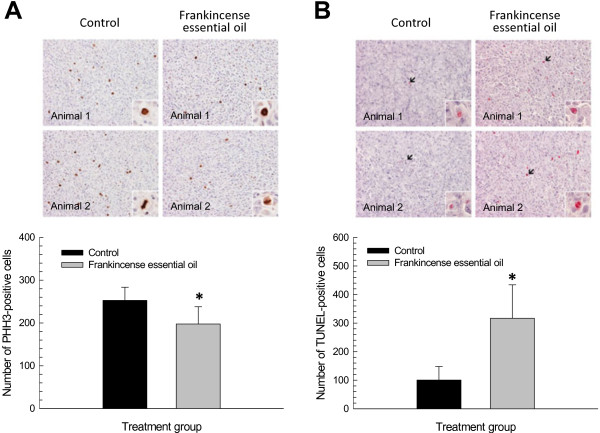
**Frankincense essential oil-induced anti-tumor activity in a xenograft mouse model.** Harvested tumors were subjected to formalin fixation and paraffin embedding. Tumor blocks were cut at 5 μm thick, deparaffinized, rehydrated, and stained for cell growth and death markers. (**A**) Tumor cell growth. Tumor cells that are actively proliferating at the time of harvest were detected by PHH3 immunohistochemical staining. (**B**) Tumor cell death. Tumor cells undergoing apoptosis at the time of harvest were assessed by TUNEL staining. Images from 2 of each control and frankincense essential oil treated animals are presented (20x magnification); and inserts were taken at 60x magnification to highlight positive immunoreactivity. Immunoreactive cells were counted from a total of 20 randomly selected fields at 40x magnification from 2 separate sections from each animal. Results are presented as total numbers of PHH3- and TUNEL-positive tumor cells from these fields. * and ** indicate statistical differences between untreated and essential oil-treated groups at *P* < 0.05 and *P* < 0.01, respectively.

Immunohistochemistry for PHH3 recognizes mitotic figures from early prophase through metaphase, anaphase, and telophase on the condensed chromatin. This method provides a quantification of mitotic activities in tumor cells [23]. Numbers of PHH3-immunoreactive cells were significantly lower in frankincense essential oil-treated tumors (197.5 ± 40.4) than in the control group (252.8 ± 30.6) (Figure 6A). By labeling the terminal end of nucleic acids, TUNEL is a quantitative method to determine cells undergoing apoptosis [24]. Significantly higher numbers of TUNEL-positive cells were detected in the experimental group receiving frankincense essential oil (316.7 ± 117.3) than the control group (100.6 ± 47.2) (Figure 6B).

## Discussion

Gum resins harvested from *Boswellia* species have long been used in Ayurvedic and traditional Chinese medicine to treat a variety of health issues. In addition, frankincense has many pharmaceutical uses particularly in anti-inflammatory activity. In the present communication, we studied anti-tumor activity of frankincense essential oil hydrodistilled from *Boswellia sacra* gum resins against a panel of human pancreatic cancer cell lines. We demonstrated that frankincense essential oil is highly effective in suppressing proliferation and inducing cytoxocity of various pancreatic cancer cell lines; and these *in vitro* activities correlate with the repression of cell cycle regulators and activation of the caspase pathway. Consistent with the *in vitro* activities, frankincense essential oil was effective in reversing tumor growth, suppressing tumor cell growth, and inducing tumor cell death in a heterotopic xenograft human pancreatic cancer mouse model.

The abundance of high molecular weight compounds is positively correlated with distillation time and temperature, based on GC-MS and HPLC analyses. Longer duration and higher temperature distillations produce greater quantities of high molecular weight compounds (sesquiterpenes and boswellic acids). The presence of high molecular weight compounds might be positively correlated with frankincense essential oil-induced cytotoxity in human pancreatic cancer cells. First, Fraction IV is more potent than Fraction I, II, or III essential oil in suppressing tumor cell growth and inducing tumor cell death. Second, differential fractionations of Fraction IV essential oil enriched with high molecular weight compounds, when *alpha*-pinene contents were lowered to 5-10%, were significantly more potent than Fraction IV essential oil in inducing tumor cell death (data not shown). Results from cell viability and cell cytotoxicity assays suggest that high molecular weight compounds and/or ratios of these compounds present in Fractions III and IV essential oils play a significant role in anti-tumor activity. However, due the complexity of chemical constituents in essential oil, the active compound(s) responsible for anti-tumor activity has not been identified. Identification of active component(s) in frankincense essential oil and its activated molecular pathways in inducing tumor cell death will be a subject of future studies.

Boswellic acids have been suggested as a major component in frankincense for the observed anti-tumor activities. For example, ethanol extracts of *Boswellia serrata* gum resins contain a defined amount of boswellic acids, and have cytotoxic and pro-apoptotic activities against leukemia cell lines (HL-60, K 562, U937, MOLT-4, and THP-1) and brain tumor LN-18 and LN-229 cells [25]. Purified boswellic acids exhibit potent cytotoxic activities against cultured human neuroblastoma cell lines (IMR-32, NB-39, and SK-N-SH) [26], and inhibit DNA, RNA, and protein synthesis in human leukemia HL-60 cells [27]. Furthermore, boswellic acids including acetyl-11-keto-β-boswellic acid (AKBA) have been shown to possess anti-tumor activity against a variety of human cancer cell lines including meningioma cells [28], leukemia cells [27,29], hepatoma cells [30], melanoma cells, fibrosarcoma cells [31], colon cancer cells [32,33], prostate cancer cells [34,35], and pancreatic cancer cells [36,37] in both *in vitro* and *in vivo* conditions.

However, boswellic acids may not be the only compounds in frankincense essential oil for inducing pancreatic cancer cell death. Total boswellic acids contents were not proportionally related to essential oil-induced tumor cell cytotoxicity among different fractions by comparing Tables 2 and 3. Additionally, frankincense hydrosol, the aqueous distillate of hydrodistilled *Boswellia sacra* gum resins, contained 0.0 to 15.5% boswellic acids, but did not have detectible cytoxicity against tumor cells even when a 1:5 dilution was added to the cultures (data not shown). Finally, tirucallic acids purified from *Boswellia carteri* gum resins have been shown to induce human prostate cancer cells death [38]. We also observed that frankincense essential oil enriched with high molecular weight compounds but lower boswellic acids contents compared to Fraction IV essential oil was much more potent at inducing cytotoxicity in cultured pancreatic cancer cells (data not shown). With the complexity and mixture of chemical compounds in frankincense essential oil, our results are in agreement with other reports that crude extracts of frankincense are more potent than boswellic acids alone in inducing cytotoxicity in malignant cells [25]. The higher potency of total extracts, not just boswellic acids, may result from a combination of multiple active compounds.

Frankincense essential oil-regulated cell cycle regulators and signaling pathways were compared to boswellia acids-activated pathways in a variety of cancer cell lines. It has been reported that boswellic acids can regulate tumor cell viability by activating a variety of mechanisms. AKBA arrests cancer cells at the G1 phase of cell cycle, suppresses levels of cyclin D1 and E, cdk 2 and 4, and Rb phosphorylation, as well as increases expression of p21 through a p53-independent pathway [33,39]. AKBA activates death receptor-5 through elevated expression of CATT/enhancer binding protein homologus protein in human prostate cancer LNCaP and PC-3 cells [35]. Boswellic acids including AKBA strongly induce apoptosis through activation of caspase-3, -8, and −9 and cleavages of PARP in colon cancer HT29 cells and hepatoma HepG2 cells [30,32]. In addition, AKBA inhibits topoisomerases I and II without inhibiting DNA fragmentation in glioma and leukemia HL-60 cells [40,41]. Our results demonstrated that frankincense essential oil suppresses cyclin D1 and cdk4 proteins expression in pancreatic cancer cells. Cyclins function as regulators of CDK kinases; cyclin D1 forms a complex with and functions as a regulatory subunit of cdk4, this activity is required for G1/S transition in cell cycle [42,43]. Frankincense essential oil suppressed cyclin D1 and cdk4 expression may lead to suppressed Rb phosphorylation which results in suppressed cell cycle progression in pancreatic cancer cells [44]. Consistent with results from boswellic acids-treated HT29 and HepG2 cells, essential oil-induced apoptosis in pancreatic cancer cells is caspase-dependent based on the cleavages and activation of caspase-3, -8, -9, and PARP in these cells.

Boswellic acids have also been shown to activate multiple signaling pathways in human cancer cells. Boswellic acids and AKBA activate the PI3K/Akt pathway in colon cancer HT29, HCT-116, SW480, and LS174T cells [45]. Although AKBA has been reported to rapidly and potently inhibit the phosphorylation of Erk1/2 in primary cultures of meningioma cells [28], other studies show that boswellic acids and AKBA activate Erk1/2 in human polymorphonuclear leukocytes and platelets [46,47]. Our results are in line with these reports that frankincense essential oil increases levels of Akt phosphorylation at Ser(473) and enhances Erk1/2 activation in most or all four pancreatic cancer cell lines. Activation of Akt and Erk1/2 signaling molecules in cancer cells by anti-cancer compounds with pro-apoptotic activity have been reported [48,49]. Biological activities and significances of the transient activation of PI3K/Akt and Erk1/2 pathways by frankincense essential oil in inducing pancreatic tumor cell death require further studies.

Boswellic acids can activate additional pathways in cancer cells. For example, boswellic acids can inhibit nuclear factor-κB and STATs activities in tumor cells [36,50]. In addition, Park *et al*. reported that AKBA down-regulates the expression of COX-2, MMP-9, CXCR4, and VEGF [36]. Pathways that are activated by a mixture of chemical components in frankincense essential oil are expected to be more complicate than the results presented in this communication. We reported that frankincense essential oil simultaneously modulates the activation of multiple signaling pathways and expression of multiple genes related to negative regulation of cell proliferation and cell cycle progression, as well as positive regulation of apoptosis in human bladder cancer J82 cells [10].

Both heterotopic and orthotopic xenograft human cancer mouse models have been used to study anti-tumor activity of boswellic acids *in vivo*. Administration of boswellic acids have been shown to significantly suppress the progression of brain tumor [7], prostate cancer [34,39], and pancreatic cancer [36]. In our heterotopic xenograft pancreatic cancer mouse model, wide variations of tumor growth were observed before the initiation of treatment in both groups receiving PBS (97–174 mm^3^) and frankincense essential oil (96–197 mm^3^). The wide variation of tumor growth provides an explanation why tumor weight is not statistically significant between these two groups. In addition, the duration of the present study is shorter than other reports using boswellic acids or AKBA in xenograft animal models [34,36,39]; a more frequent and longer treatment regime might further enhance the differences between the control and experimental groups. In agreement with frankincense essential oil-induced DNA fragmentation in cultured pancreatic cancer cells, there are elevated numbers of TUNEL-positive tumor cells in the essential oil-treated group. Since apoptosis is observed within hours following *Boswellia sacra* Fraction IV essential oil treatment in cultured cancer cells, frankincense essential oil-activated anti-proliferative and pro-apoptotic activities may be more prominent if tumors were studied shortly after essential oil administration.

Frankincense has been used and investigated as a cancer therapeutic agent in clinical settings. For examples, frankincense gum resins are used as a main component in an anti-cancer drug in traditional Chinese medicine. In addition, frankincense is considered as an alternative medicine in Arab countries and has been used as an anti-cancer agent to treat neoplastic diseases. In a prospective, randomized, placebo-controlled, double-blind clinical trial conducted in patients irradiated for brain tumors, an extract prepared from *Boswellia serrata* gum resins (H15) significantly reduces cerebral edema with anti-tumor properties, without severe adverse effects [9]. *Boswellia serrata* extracts is included as an adjuvant agent in patients with high grade gliomas under an ongoing phase II clinical trial ( http://www.cancer.gov/clinicaltrials/search/view?cdrid=445603&version=healthprofessional).

We expect that frankincense essential oil obtained from hydrodistillation of *Boswellia sacra* gum resins can be a novel and alternative therapeutic agent to suppress pancreatic cancer progression and metastasis. Nevertheless, a standardized procedures to process *Boswellia* species gum resins and to prepare essential oil are required for providing consistent anti-tumor activity. Although the identification of active compound(s) responsible for essential oil’s anti-cancer activity would be important, it may not be necessary if a standard assessment of chemical compositions and predicted biological functions can be established. Although no serious safety and toxicity issues have been raised in animal models and patients receiving oral administration of frankincense extracts [51-55], the maximum safe dose of essential oil needs to be defined for cancer therapy, and pharmacokinetics and pharmacodynamic properties of essential oil need to be determined.

## Conclusion

Frankincense essential oil prepared from hydrodistillation of *Boswellia sacra* gum resins activates anti-proliferative and pro-apoptotic activities in human pancreatic cancer cells in cultures and reverses tumor growth in a heterotopic xenograft mouse tumor model. The anti-tumor activity of frankincense essential oil is mediated through multiple signaling pathways and cell cycle regulators, and is dependent upon caspase pathway-mediated apoptosis. Based on the safety and tolerability of different frankincense extracts in animal models and human subjects, extracts from hydrodistillation of *Boswellia sacra* gum resins might represent a new therapeutic option on pancreatic cancer treatment in human patients after efficacy can be further confirmed. In addition, pharmacokinetics and pharmacodynamic studies are required to optimize the administration frankincense essential oil for anti-cancer therapy.

## Abbreviations

AKBA: Acetyl-11-keto-β-boswellic acid; GC: Gas chromatography; LDH: Lactate dehydrogenase; MS: Mass spectrometry; HPLC: High-performance liquid chromatography; PARP: Poly-(ADP-ribose)-polymerase; PHH3: Phospho-histone H3; TUNEL: Terminal deoxynucleotidyl transferase dUTP nick end labeling.

## Competing interests

CW and GY are affiliated with Young Living Essential Oils. The rest of authors declare that they have no competing interests.

## Authors’ contributions

XN carried out xenograft animal studies. MMS, CW, and GY prepared and analyzed frankincense essential oils prepared from *Boswellia sacra* gum resins. QY, AC, and HKL performed molecular and cell biology studies in cultured pancreatic cancer cells. KMF studied pathological presentations of tumor cells from xenograft mice. MMS, KMF, RGP, CW, GY, JZ, and HKL conceived the idea, designed the experiments, and interpreted the experimental results. All authors contributed to manuscript preparations and approved the final manuscript.

## Pre-publication history

The pre-publication history for this paper can be accessed here:

http://www.biomedcentral.com/1472-6882/12/253/prepub
